# Biochemical testing and pathology reveal rare cause of pediatric acute liver failure: Hyperornithinemia‐hyperammonemia‐homocitrullinuria syndrome

**DOI:** 10.1002/jpr3.70206

**Published:** 2026-07-27

**Authors:** Tierra L. Mosher, Kathryn S. Czepiel, Matthew B. Neu, Andrea Carolina Cortes Fernandez, L. Walden Browne, Annie D. Niehaus, Roberto Gugig

**Affiliations:** ^1^ Division of Gastroenterology, Hepatology and Nutrition, Department of Pediatrics Stanford University School of Medicine Palo Alto California USA; ^2^ Division of Medical Genetics, Department of Pediatrics Stanford School of Medicine Palo Alto California USA; ^3^ Department of Pathology Stanford School of Medicine Palo Alto California USA

**Keywords:** metabolic, SLC25A15, urea cycle disorder

## Abstract

Hyperornithinemia‐hyperammonemia‐homocitrullinuria (HHH) syndrome is a rare metabolic condition that can cause lethargy, ataxia, tachypnea, nausea, vomiting, seizures, coma, and acute liver failure. We present a 26‐month‐old female with acute liver failure who was diagnosed with HHH 1 week after admission. Histology revealed an acute hepatitic pattern of liver injury with numerous acidophils and glycogenated nuclei without zonal distribution, the former a rarely discussed feature. Acute management included intravenous dextrose and intralipids, with chronic management requiring a protein‐restricted diet, glycerol phenylbutyrate for nitrogen scavenging, and citrulline supplementation to support the urea cycle. This case contributes to our growing understanding of the phenotypic presentation of HHH, a rare genetic cause of liver failure not included on newborn screening but with specific treatment implications. We also highlight the importance of collecting biochemical genetics labs and liver biopsy early in the disease course and discuss how a patient's voluntary self‐restriction of protein can offer helpful clues in the diagnostic evaluation.

## 1 INTRODUCTION

Pediatric acute liver failure (PALF) is the acute onset of liver disease and coagulopathy not corrected by vitamin K administration without evidence of chronic liver disease as described in the PALF Study Group (PALFSG) entry criteria.^1^ PALF is rare, affecting fewer than 1000 patients annually from diverse causes; metabolic disorders account for a small fraction.^1^

Hyperornithinemia‐hyperammonemia‐homocitrullinuria (HHH) syndrome (Online Mendelian Inheritance in Man [OMIM] #238970) is a rare metabolic condition characterized by neurologic and hepatic abnormalities in the setting of hyperammonemia with presentations varying from neonatal hyperammonemic crises to later‐onset neurocognitive deficits, liver dysfunction, and acute encephalopathy.^2^ We report a 26‐month‐old female with PALF whose timely biochemical workup and liver biopsy enabled a diagnosis of HHH and initiation of targeted therapy.

## 2 CASE REPORT

A 26‐month‐old female with no past medical history was transferred to a quaternary care center after presenting with lethargy, reduced oral intake, and frequent emesis. Initial labs showed platelets 428, monocytes 2, chloride 113, carbon dioxide (CO_2_) 19, aspartate transaminase (AST) 125, alanine transaminase (ALT) 255, and procalcitonin 1.77. Infectious studies were positive for coronavirus (not Covid‐19) and Ebstein–Barr virus (EBV) but negative for influenza, respiratory syncytial virus (RSV), rhinovirus/enterovirus, and adenovirus. Her transaminemia was attributed to viral illness, and the patient was admitted to the general pediatric inpatient service for intravenous (IV) fluids to correct presumed dehydration. Her mental status improved initially after receiving IV fluids, but she soon became fussy, fatigued, and demonstrated reduced oral intake. Labs demonstrated worsening liver injury with AST 4323, ALT 3830, alkaline phosphatase (ALP) 481, gamma‐glutamyl transferase (GGT) 49, international normalized ratio (INR) 3.1, and ammonia 219. The hepatology service was consulted, and the patient was transferred to the pediatric intensive care unit (PICU) for management and evaluation of presumed PALF.

**Figure 1:**
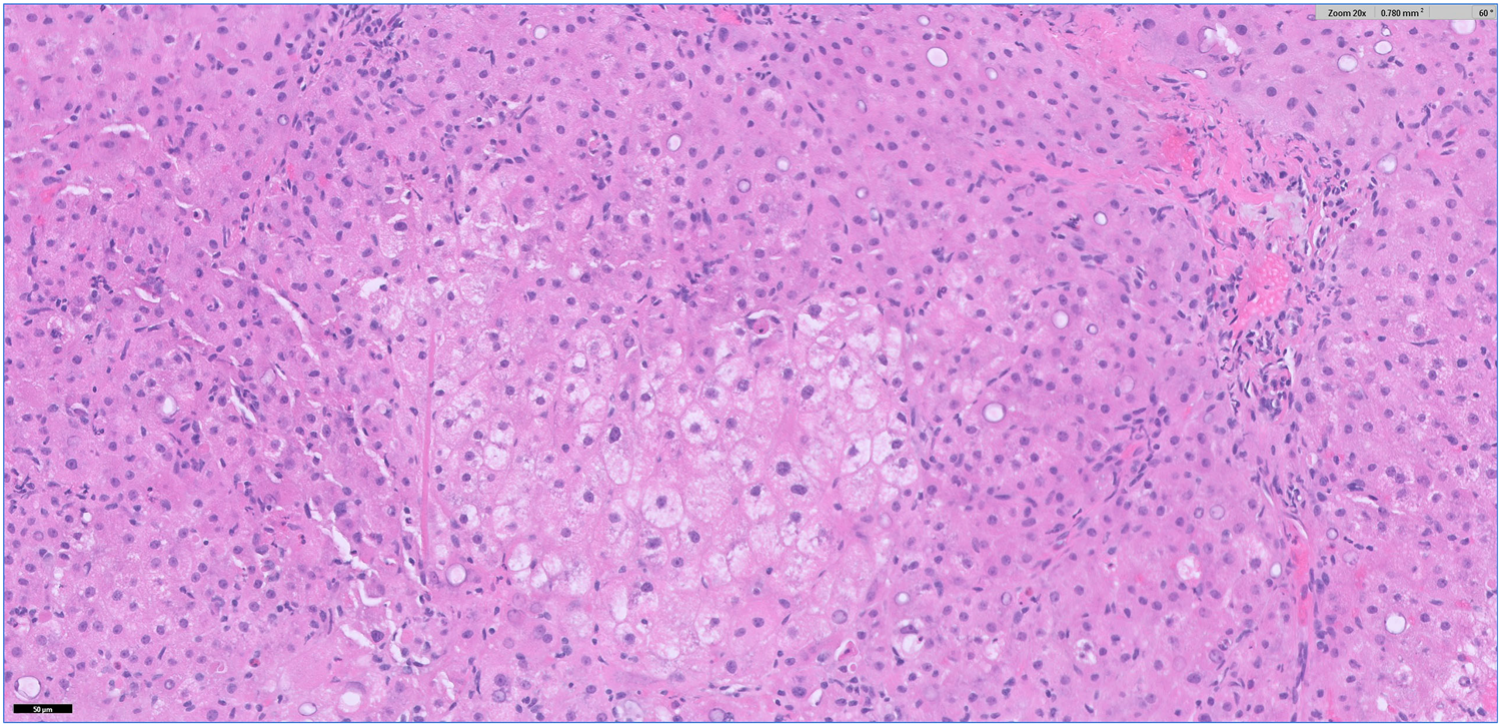
Liver biopsy histology. The liver shows numerous scattered necrotic hepatocytes without a significant increase in inflammation. Several scattered hepatocytes with glycogenated nuclei are present without a clear‐cut specific zonal distribution. Several hepatocytes also show reactive changes characterized by foamy cytoplasm (H&E section). An irregular perisinusoidal and patchy septal pattern of fibrosis was present on the trichrome stain (not shown). H&E, hematoxylin and eosin.

In the PICU, the patient appeared encephalopathic with fluctuating mental status alternating between long periods of sleepiness and hyperactivity. We initiated a broad work‐up for PALF including collection of plasma amino acids, acylcarnitine profile, and a liver biopsy. Histology demonstrated moderate hepatitis and scattered hepatocytes with glycogenated nuclei (Figure 1). IV dextrose‐containing fluids and ammonia‐lowering therapy with rifaximin and lactulose reduced ammonia to 65 (ref range 11–35 μmol/L) by Day 3. A transient hyperammonemic spike (182 μmol/L) on Day 4, associated with brief jerking motions with eye fluttering during sleep, was investigated with continuous electroencephalogram, which ruled out seizure activity, and the hyperammonemia resolved with medical therapies without the requirement of dialysis. She transferred to the inpatient floor service on Day 5.

**Figure 2:**
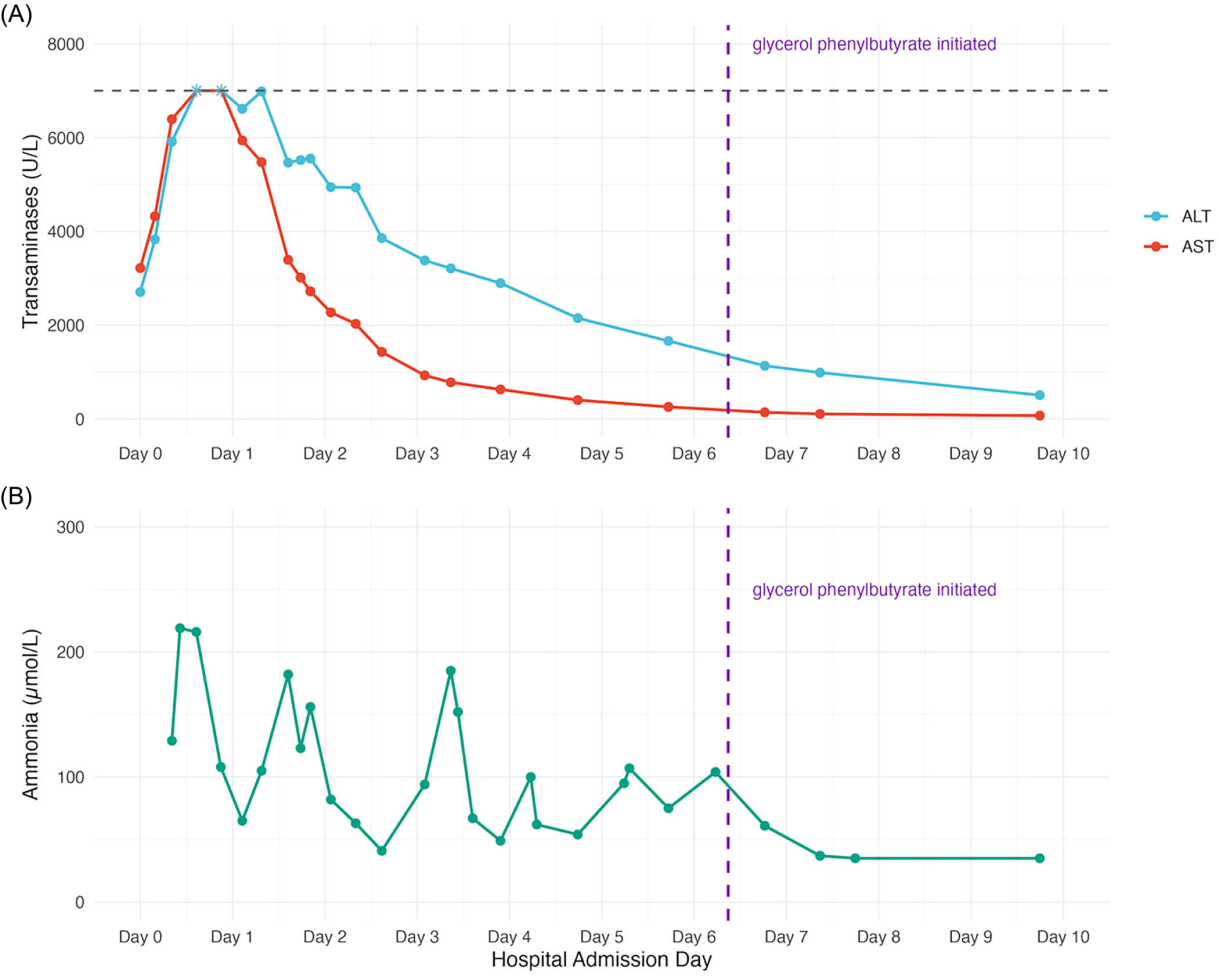
Laboratory trends throughout hospital course. Levels of ALT (blue) and AST (red) are shown in (A). AST and ALT values at 7000 U/L represent the laboratory's upper reportable limit, with a normal reference range of <77 (U/L, AST) and 0–35 (U/L, ALT). Ammonia levels are shown in (B). The laboratory's normal reference range for ammonia is 11–35 μmol/L. Day of admission is shown on the *x*‐axis and laboratory values on the *y*‐axis. ALT, alanine transaminase; AST, aspartate transaminase.

Despite clinical recovery, ammonia remained mildly elevated (Figure 2). On Day 8, plasma amino acids revealed elevated ornithine (495 nmol/mL, ref 10–163 nmol/mL) and homocitrulline (6 nmol/mL, normally undetectable). Urine amino acids and urine organic acids were notable for unquantified presence of homocitrulline and lactic and orotic aciduria, respectively. This workup and clinical features revealed a diagnosis of HHH. In addition to restarting IV dextrose‐containing fluids, the consulting medical genetics team recommended IV intralipids, low‐protein diet, citrulline supplementation, and glycerol phenylbutyrate. Family history revealed voluntary protein avoidance at home; for example, she avoided high‐protein foods (meats, eggs, cheese, and cow's milk) as intake often prompted vomiting. Within days of treatment initiation, the patient's clinical status, ammonia levels, and liver labs improved, and she was discharged (Figure 2).

Clinical single gene testing of *SLC25A15* was performed on peripheral blood by a large clinical genetic testing laboratory, licensed by the College of American Pathologists (CAP) and accredited by Clinical Laboratory Improvement Amendments (CLIA), using in‐house massively parallel (“next‐generation”) sequencing technology. This testing identified a homozygous pathogenic variant in *SLC25A15* (c.22C>T, p.Gln8*) anticipated to cause loss‐of‐function consistent with HHH. This variant has previously been observed in the compound heterozygous state in an individual with HHH.^3^ Familial testing, including both parents and three siblings, was recommended. Since discharge, the patient remains mildly neurodevelopmentally delayed but clinically well without further hyperammonemic episodes on her current therapy and low‐protein dietary regimen.

## 3 DISCUSSION

**Figure 3:**
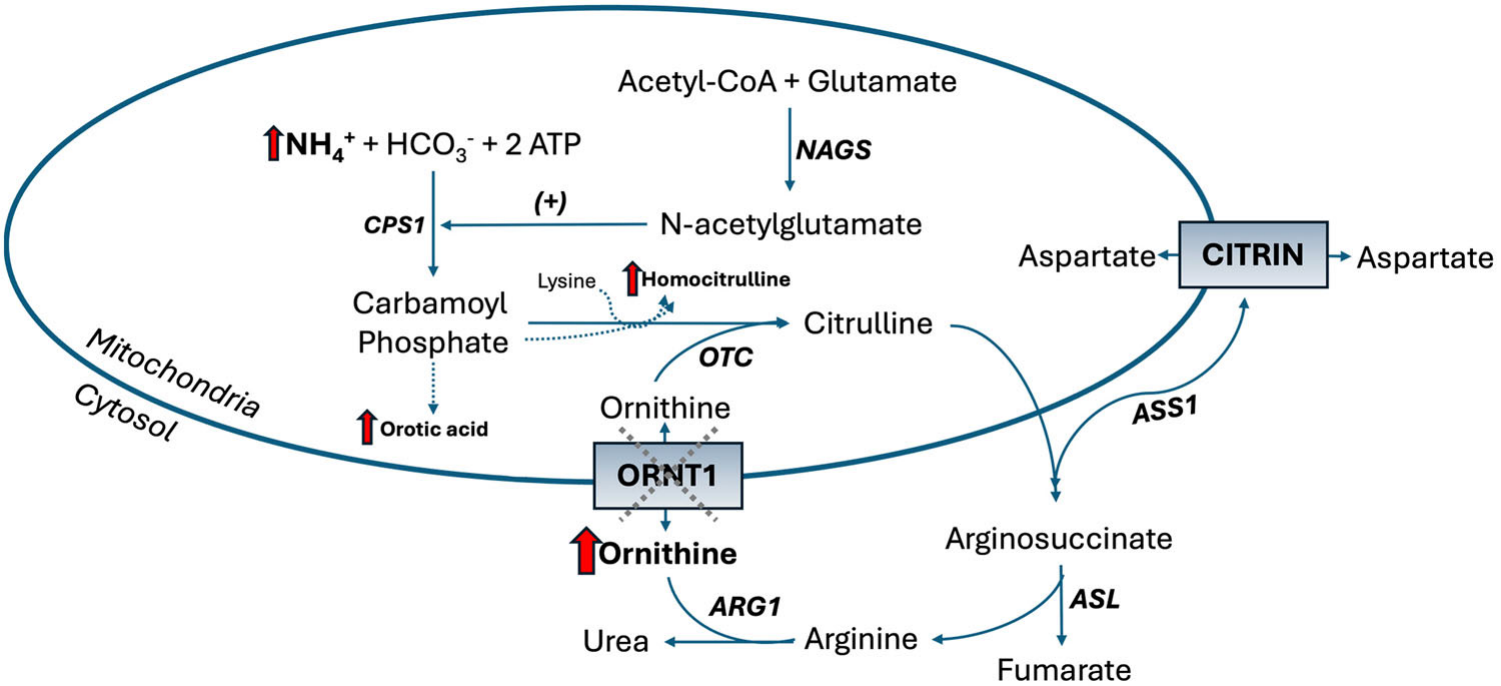
Urea cycle diagram. The ORNT1 transporter (gray dashed “X”) is encoded by the *SLC25A15* gene and is responsible for allowing ornithine to enter the mitochondria. Once in the mitochondria, ornithine continues within the urea cycle to generate citrulline. The ORNT1 transporter is dysfunctional in individuals with HHH syndrome, which causes elevated plasma ornithine levels (red arrow). Underused carbamoyl phosphate either reacts with lysine to form homocitrulline or enters the pyrimidine pathway to form orotic acid. Its underutilization also leads to an excess of ammonia. Consequently, their accumulation leads to increased serum ammonia and urinary excretion of homocitrulline (homocitrullinuria) and orotic acid. Figure modified from Figure 1 in Simpson et al.^4^ ATP, adenosine triphosphate; ASL, argininosuccinate lyase; HHH, hyperornithinemia‐hyperammonemia‐homocitrullinuria; NAGS, N‐acetylglutamate synthase; OTC, ornithine transcarbamylase.

HHH is an autosomal recessive condition caused by biallelic pathogenic variants in the *SLC25A15* gene. *SLC25A15* encodes ORNT1, an ornithine‐citrulline antiporter protein responsible for transporting ornithine across the inner portion of the mitochondrial membrane.^2^ Impaired ornithine transport leads to accumulation of ornithine and lysine in the bloodstream, which then disturbs the urea cycle, leading to overaccumulation of nitrogen and ultimately hyperammonemia (Figure 3). Carbamylation of excess lysine in the bloodstream leads to increased presence of its side product homocitrulline.^2^ Clinically, HHH manifests as developmental delay, motor dysfunction, and seizures chronically, and acutely with lethargy, ataxia, tachypnea, nausea, vomiting, seizures, coma, and liver failure.^2^ There is higher prevalence of HHH in founder variant regions such as Canada, Asia, and Europe, but this disease is rare, with fewer than 200 cases documented worldwide.^2^, ^3^

Typical management involves lowering nitrogen to reduce production of ammonia via a combination of restricting protein, replacing citrulline to allow for continuation of the urea cycle, and nitrogen scavengers, such as glycerol phenylbutyrate.^5^, ^6^, ^7^

Liver biopsy in HHH is nonspecific, with prior reports ranging from histologically “normal” to marked perinuclear glycogenated nuclei and vacuolization.^8^, ^9^ The histologic findings in any kind of urea cycle deficit are similar to those of glycogenic hepatopathy (usually due to Type 1 diabetes mellitus), glycogen storage disease, as well as Wilson disease. Our patient's biopsy showed the unusual finding of a hepatitic pattern of injury characterized by numerous scattered acidophils (necrotic hepatocytes) without a commensurate increase of an inflammatory infiltrate, as well as numerous hepatocytes with prominent glycogenated nuclei without a zonal distribution, the latter of which is the usual expected finding for a urea cycle deficit. Additionally, there were patchy areas of hepatocytes with reactive changes characterized by foamy cytoplasm. At the time of the biopsy, Wilson Disease, which also may have a quite variable spectrum of liver biopsy findings, was considered in the preliminary histologic differential diagnosis. A rhodanine copper stain was negative, and the patient's ceruloplasmin was in the normal range; while these two tests are not completely sensitive, the results do not support a diagnosis of Wilson disease.

## 4 CONCLUSION

Our case highlights a case of PALF followed by an atypical recovery pattern notable for persistent hyperammonemia despite resolved coagulopathy and improved transaminemia found to be secondary to HHH. One important takeaway is the importance of biochemical labs in any patient with PALF without clear etiology on labs, imaging, or histology. Additionally, it is important to obtain thorough past medical and dietary histories during patient intake and maintain a broad differential in any patient with atypical recovery from PALF to ensure this rare condition is considered. Finally, liver histology interpretation must integrate nonspecific findings, such as glycogenated nuclei, to guide metabolic evaluation.

## CONFLICT OF INTEREST STATEMENT

The authors declare no conflicts of interest.

## ETHICS STATEMENT

The patient's mother has provided written informed consent to write and publish the de‐identified case presentation of their child as above.
